# Coping with singleness

**DOI:** 10.1186/s40695-023-00086-1

**Published:** 2023-02-21

**Authors:** Shakiba Pourasad Shahrak, Serge Brand, Ziba Taghizadeh

**Affiliations:** 1 Department of Midwifery and Reproductive Health, School of Nursing and Midwifery, Tehran University of Medical Sciences, Tehran, Iran; 2 Center for Affective, Stress and Sleep Disorders (ZASS), Psychiatric Clinics (UPK), University of Basel, 4002 Basel, Switzerland; 3 Division of Sport Science and Psychosocial Health, Department of Sport, Exercise, and Health, University of Basel, 4052 Basel, Switzerland; 4 Substance Abuse Prevention Research Center, Health Institute, Kermanshah University of Medical Sciences, Kermanshah, 67146 Iran; 5 Sleep Disorders Research Center, Kermanshah University of Medical Sciences, Kermanshah, 67146 Iran; 6 School of Medicine, Tehran University of Medical Sciences, Tehran, 25529 Iran; 7 Nursing and Midwifery Care Research Center, and Department of Midwifery and Reproductive Health, Tehran University of Medical Sciences, Tehran, Iran

## Abstract

**Background:**

The number of never-married women is increasing worldwide. According to a recent census (2016) this trend is also apparent in Iran. The aim of the present study was to investigate how never-married Iranian women cope with their single status.

**Methods:**

The present study was qualitative in nature. Purposeful sampling with maximum variation was used to select 18 never-married women aged over 35. Data were analyzed on the basis of conventional content analysis and inductive reasoning.

**Results:**

One hundred fifty-four codes, nine subcategories, three categories, and one theme were extracted. The three categories were: (1) responding to sexual needs (sub-categories: having sex; masturbation; sexual abstinence); (2) responding to emotional needs (sub-categories: getting used to being alone; living with family; closer relationship with good friends); (3) lifestyle changes (subcategories: accepting God's destiny; striving for beauty and health; becoming absorbed in work and education).

**Conclusions:**

Results showed that never-married women aged over 35 tried to adapt to sexual and emotional needs and lifestyle changes as proxies of singleness in various ways. It appears that these women adopted several strategies to cope with the lack of a spouse, children, or family life, these normally being developmental tasks characteristic of early adulthood.

## Background

Toward the end of the last century, the phenomenon of globalization caused significant changes in social and cultural norms throughout the world. Among others, this phenomenon appeared to result in an increase in the proportion of never-married women [1]. Estimates show that the number of single women has increased worldwide [2]. In the United States, the number of never-married women over the age of 35 increased by about 10% between 1980 and 2010 [3]. According to the most recent national censuses conducted across the whole of Iran, the number of never-married women, was 89,402 in 1986, and 789,544 by 2016. The ratio of never-married to married women was 0.005 in 1986; it had increased to 0.024 in 2016 [4]. The following factors appeared to be causally linked to the increase in the proportion of never-married women [5]: improvements in social support, access to health care services, guarantees of reproductive and sexual rights, having property, wider cultural and religious acceptance of singleness, a greater tendency for those in the higher socio-economic groups to remain single, and finally a reduction in the numbers of employed and well-off men. Jones [6] argued that higher level of education and having a job were two important factors in a change in marital practice, effectively a delay in the age of marriage. In Southeast Asian countries, other reasons for delayed marriage include rising divorce rates, disincentives to have children, and marriage [6]. However, the concept of marriage has changed dramatically [7], and results of this change include a delay in age of marriage or an increase in the number of older people who have never-married [8].

In one study, a never-married woman was defined as one who “has never married, is over 30 years old, is not cohabiting and has no child” [9]. Another study defined a never-married woman as an “old maid” who could not get a man because she is unattractive, handicapped, or incompetent or a woman who lives in a city and does not want a man because she is highly educated, ambitious, single-minded, determined, active or with high social class [7].

Considering the concept from a developmental psychological perspective [10], the time-period of early adulthood is critical: in addition to becoming economically, emotionally, cognitively, and socially independent from the family of origin, establishing a stable and long-term relationship, including marriage, family planning, and giving birth to children, and working towards educational achievements and employment opportunities are among the key developmental tasks of this developmental stage. Note that these developmental tasks are normative in so-called collectivistic societies (70% of all current societies), including Iran, while individualistic societies (the remaining 30%) emphasize a more individually-tailored course of development [11].

Marriage can be an important source of emotional and personal development [12]. In eastern communities, marriage is highly valued both socially and culturally. Consequently, women who are single face stresses and challenges in their lives [13]. Meeting these challenges places a reliance on the coping capacities of never-married women. Although single women can develop through education and employment [14] and may be satisfied with their single status [15], they may face the prospect of loneliness in old age and must prepare themselves to adapt to this prospect [16].

The transactional theory of coping postulates that coping is an evolving process that changes in response to context and in an effort to manage different internal and external demands [17]. Accordingly, the transactional theory of coping assumes that successful coping involves an ability to adjust and alter coping strategies in ways that facilitate positive outcomes and a capacity to modify coping behavior to meet the demands of current stresses [18].

Regarding the increasing pattern of never-married women in Iran and the world and limited researches on this issue, and note that there is a particular lack of research into the coping strategies of never-married women, the aim of the researchers was to investigate how never-married Iranian women over 35 seek to cope with their psychological and social situation. We hope to draw the attention of the country's policymakers and health planners so that the country may be better prepared to address the needs of this group.

## Methods

### Study design

In this qualitative study, we employed conventional content analysis and inductive reasoning since there is limited research on never-married women and their coping with singlehood in the Iranian context.

### Participants

Participants were 18 never-married women between the ages of 36 and 64. Participants had never been married, legally or illegally. They were selected through purposeful and snowball sampling to achieve maximum variation with respect to age, level of education, occupation, and residential circumstances (living with family members or alone). Participants were eligible for inclusion if they did not report having a mental illness based on self-declarations and were heterosexual. They were selected from cities in Iran representing different cultural backgrounds.

For sampling, at first, the researcher identified and invited the never-married women meeting inclusion criteria who were working in her school to take part in the study. After that, for access to more participants with more maximum variation, she asked participants to introduce other never-married women between their friends and relatives. On the other hand, the researcher started looking for never-married women among her own relatives and friends, and also she went to religious places that many people, as well as never-married women, go to pray.

### Data collection

In this study, the number of participants was 18 though five were interviewed twice. Consequently, we had 23 interviews with these 18 participants. This arose because two participants in the study did not have enough time to complete the interview in the first session, therefore we obtained their consent to call them for a second appointment to finish the interview. However, because we probed into participants' private life, some of them spoke incoherently about certain topics, and occasionally, after transcription of some of the interviews, it became clear that the deeper meaning of some participants' statements could not be ascertained without additional information, necessitating another interview. In this case, the researcher contacted the participant, arranged another interview time, and interviewed them again. Before the interview, the researcher gave details about the goals of the study and informed participants about the confidentiality of information and data. Also, the researcher explained about the voluntary participation and freedom to leave the interview whenever they want. The interviews started after obtaining informed consent from participants. The location of the interview was selected according to participant's wishes such as their workplace, university, or park. Data were collected from December 2019 to March 2020.

Due to the sensitive nature of our research, we made a great effort to communicate well with our participants. We worked hard to gain their confidence. Before the interview, we gave them information about the study's objectives and allowed them to ask any questions they might have. We asked them to share their life stories and how they dealt with being single after getting their informed consent. To obtain a rich picture of the phenomena, researchers conducted in-depth, semi-structured, and face-to-face interviews at different times and places using guiding questions such as “How did you cope with your celibacy?” and “Can you talk about your lifestyle?”. During the interview, the researcher asked exploratory questions such as 'Can you explain this in more detail?' To recruit participants, the principal interviewer frequented workplaces, universities, and parks, and invited potential participants to take part in the study. Interviews lasted between 45 and 60 min and continued until data saturation. In qualitative research, the determination of sample size is contextual and partially dependent upon the scientific paradigm under which investigation is taking place [19, 20]. In this study, sampling continued until data saturation. A saturation point was reached when no new data or category emerged by the 23rd interview. The language of the interviews were Persian. All interviews were recorded.

### Data analysis

MAXQDA-10 software was used for data management. Data analysis was performed concurrently with data collection using the Graneheim and Lundman [21] method. Conventional content analysis is usually used when knowledge about a phenomenon is limited. In the present case, the researchers allowed the formation of codes and categories to emerge from the actual research data instead of using codes predetermined on the basis of theory. In this study, each interview was transcribed and analyzed as soon after the interview as possible, for example on the day of the interview or the day following recording. After full transcription, the text was read line by line, semantic units were specified and codes were extracted. Then data reduction took place. Finally, subcategories and categories of analysis emerged from these codes.

### Rigor

To ensure the trustworthiness of the data [22], we used the Guba and Lincoln (1994) method with the following key criteria of credibility, dependability, transferability, and conformability. The codes were reviewed, and participants confirmed the accuracy of the codes to guarantee the credibility of the research. An external supervisor ensured the dependability of the study. Other unmarried women not involved in the study confirmed the transferability of the results. Both a coding process and peer reviews ensured the conformability authenticity.

Also, data triangulation increases the rigor of data. It can be reached by collecting data at different times and settings and by using different sampling protocols. In the present study, data triangulation was obtained by using two methods of data sampling (purposeful sampling and snowball sampling).

### Ethics approval and consent to participate

This study was a part of a Ph.D. dissertation on reproductive health. The ethical standards applied were those of the seventh and current [23] version of the Declaration of Helsinki. The present study was approved by the Research Ethics committee of the Tehran University of Medical Sciences (Ethics Code: IR.TUMS.VCR.REC.1398.420, 07/17/2019). All participants provided written informed consent prior to participation.

## Results

In this study, the age of participants was 36 to 64. They had different jobs such as running a household, clerk, teacher, academic member, doctor and one of them was without job. Most of the participants lived with their family and just four women lived alone. The participants were chosen from 12 different cities of Iran which have different cultures and backgrounds.

Sixteen out of 18 participants stated that they did not want to be single, but remained never-married because of their living situation, family, and community circumstances. In fact, they remained unmarried against their wishes. Like women who willingly remained single, they tried to cope with their conditions of life.

Totally, 154 codes, nine subcategories, three categories, and one theme were extracted from the interviews; these are shown in Table 1.

**Table 1:** Subcategories, categories, and theme extracted from the interviews

Subcategories	Categories	Theme
Having sex	Sexual issues	Coping with singleness
Masturbation		
Abstinence		
Getting accustomed to being alone	Emotional needs	
Living with family		
Closer relationship with friends		
Accepting God's destiny	Lifestyle	
Striving for beauty and health		
Becoming absorbed in work and education		

### Sexual issues

This category comprises three subcategories: having sex, masturbation, and abstinence. While religious recommendations in Islam (the religious of the most Iranian) do strongly discourage any kind of sexual activity when unmarried, not all never-married women pay attention to this prohibition. One participant said that having sex with a boyfriend was one way to meet sexual needs. *“Well, the sexual need is a strong desire in a person. I have the feeling of a married woman with my boyfriend. I feel no lack in life.”* (43 years old).

On the other side, another participant stated: *“Although I cannot have sex because of my religious beliefs, I agree with masturbation because at least, with this act, my body estrogen lasts longer and my beauty and youth are maintained.”* (37 years old).

Premarital sex is condemned in Iranian culture and religion. One participant said: *“Since we have become conscious about the good and the bad things, we have always been warned about these issues; these words have grown so much inside us that we could no longer have sex.”* (48 years old).

### Emotional needs

This category comprises three subcategories: getting accustomed to being alone, living with family and closer relationship with friends. Most participants had got used to their status as single:* “I don't look at marriage as a staple food. Marriage is like a delicious dessert, which is great if there is, but if there is not, I have my staple food.”* (42 years old).

Participant number 9 considers having a job and family as the reason for not wanting to get married and said: *“Because I don't depend on anyone financially and I have a large family, so I don't feel lonely and don't need to get married.”* (41 years old).

Single women usually choose many friends and try to maintain their relationship with them. A participant said: *“A person can get in touch more with her family in life, but her parents grow old or die. So, for lonely days, I will try to keep my friends from now so I won’t be alone in the future.”* (37 years old).

### Lifestyle change

This category has three subcategories: accepting God's destiny, striving for beauty and health, and becoming absorbed in work and education.

Accepting God's destiny in all aspects of life, especially in the field of marriage, was evident in the interviews of participants. As a 52-year-old woman put it: *“God created me alone and didn't ordain marriage for me, because God intended this for me, I accept it*.”

According to the study participants, one of the most important issues in the life of never-married women above 35 is to look more beautiful. In this regard, participant number 9 states: *“Single people pay a lot of attention to their appearance. They want to look happier.”* (41 years old).

Singles pay close attention to their physical health, stated one 64-year-old participant: *“I do health check-up every year … I need to know what is going on in my body.”*

Focusing on job and education development was another aspect of women's lives. One of the participants said: *“I tried to develop myself in education and work. Now, although I am not married, I have a Ph.D. degree and a high social level.”* (45 years old).

## Discussion

The key findings of the present qualitative study were that never-married women aged over 35 reported differentiated coping patterns as a result of their status. Specifically, responses yielded three key categories: sexual issues, emotional needs, and lifestyle (for each of which subcategories were identified). The present results importantly add to the current literature in that participants’ answers covered a broad range of sexual, emotional, and lifestyle issues. As such, the variety of results also provides an illustrative portrait of how to cope with the current status of being unmarried in a creative, proactive, and constructive fashion. Results are now discussed in more detail (see also Table 1).

For coping with unmet sexual needs, two never-married women experienced sexual intercourse, some of them masturbated and others practiced sexual abstinence. This last group stated that sexual desire has diminished over time through suppression of sexual need and they have become accustomed to living without sex. Comparing these strategies with findings from other studies, it emerges that in the United States nine out of ten women have had sex outside marriage [24] while in China this is the case for 28% of women [25]. That the frequency found in the present study is so out of line with the values reported in other studies probably reflects the cultures and religious beliefs particular to different countries. In Islam, sex outside marriage is prohibited. Iranian culture forbids females from having sexual relations at any age before or outside of marriage [26]. This cultural attitude is common to most Muslim countries in South and Southeast Asia [27, 28]. While on the one hand sexual need is a strong physiological, emotional, and social desire [29–31], on the other hand, being a virgin is a value for Iranian women. Given this, never-married women sometimes masturbate while maintaining their virginity. However, according to Islam, the religion of Iranian, masturbation is also condemned. Therefore, these women regarded abstinence, i.e., not thinking about sex and avoiding exposure to sexual arousal, as a valuable way of coping. The present findings are in line with the results from studies in Muslim countries that are similar to Iran in terms of belief and religion [27, 28]. Another reason for ignoring sexual need is the passage of time, aging, and a diminishing desire for sexual intercourse due to the lack of a sexual partner. A study by Mroczeka (2013) that conducted on single women over the age of 60 found that they ignored sexual needs because they did not have anybody to have sex with; Participants also stated that this need decreased as they got older and they felt very little of this need in themselves [32].

Another important result to emerge in the present study concerned getting accustomed to being alone. Although participants were worried about their future loneliness, they stated that they would never marry until they found the right partner; this became more apparent in their lives as they got older. But the important point is that with passing time, and acquiring advanced educational credentials and developing a career, finding a suitable husband becomes difficult because these things are happening during a period of life when women would normally be chosen by men as wives. This reality is apparent in the study by Azmawati (2015) which indicated that two circumstances, having a high level of education and a high-level job, are reasons for staying single [15]. Statistics in Iran show that the odds of marriage clearly decrease for women after the age of 35 [4]. The results of another study showed that older single women were less likely to marry than younger single women [33]. This is in line with previous findings. For example, Band- Winterstein (2014) reported that women who have never been married feel comfortable and satisfied with life because they could control their lives and have more freedom in making decisions [16]. In fact, by staying single and concentrating their time and energy on themselves and away from the responsibilities of married life, they can both make progress in life and live more freely and happily. Another study, however, reported rather different reactions. Participants in that study noted that they felt sadness in life and thought they had made a mistake; they were worried about their future loneliness and wanted to have a spouse and children [34]. We note that no participants in the present study expressed self-blame or guilt- feelings.

Two of the coping strategies with emotional issues were living with family and having good friends. Indeed, in present study, all women except three reported living with their family of origin (two lived alone due to the death of parents and one due to work in another city). In addition, participants stated that having good friends made them feel cheerful and supported. Newbold (2013) reported that social and family support, along with individual characteristics, were very important to feelings of wellbeing [35]. Likewise, never-married women mentioned that family and friends were the most important people in their lives, though it should be noted that living alone is culturally and socially discouraged for women in Iran [36]. Family support also helped never-married women to overcome the hardships of a single life [14]. In the same vein, 52% of never-married women were satisfied with their relationships with family and friends. Not surprisingly, never-married women reported that family members and friends helped them to cope with personal problems [37].

Another coping strategy for being unmarried was belief in God's destiny in marriage. Typically, participants stated: “Enshaallah”, meaning “If God willing”. In the same vein, Malaysian Muslim women also mentioned God's destiny as the most important reason for not getting married, along with their preference, not finding a good prospect, having a busy life, and caring for family responsibilities [14]. Believing in God’s decisions helped Indonesian women more easily to accept their singleness and to cope to the circumstances of their lives [27]. Single women stated that believing in God helped them to be patient and happy with life’s demands, to be better adapted to living conditions, and to experience higher self-confidence. In brief, believing in God's destiny was a coping strategy in acceptance of being unmarried [28].

The majority of participants also reported going to esthetic clinics, following the world’s fashions, having annual health checks, exercising, and paying attention to the slightest changes in body and weight. This can be the result of having more free time to spend for themselves than married people. On the other hand, the prospect of being alone in times of illness and aging can provoke a feeling of worry in never-married people and can encourage them to seek more check-ups or take more exercise because they know that they don't have any children or husband to support them [38].

In contrast, single people thought themselves to be less attractive, less acceptable, more nervous, and less sociable than those who were married. In addition, they were less satisfied with their lives and wanted to change their way of life (by getting married). They also felt lonely in their social interactions and had lower self-confidence and self-esteem. In Malaysia, never-married women are addressed as “Andartu”, meaning an “old virgin”, and most of these women felt they were unfairly treated and stigmatized [14]. Likewise, in Iran, adult never-married women are addressed as “Torshideh”, meaning “the expired woman”. In the face of such stigmatization, it appears that unmarried women are always preoccupied about losing their charm and beauty. Thus, paying attention to beauty and health appeared to be a strategy to improve self-confidence and social acceptance, and to counterbalance natural aging and possible stigmatization.

Involvement in a career or education was another reported coping strategy. In Malaysia, 70% of single women had higher education [14]. In the last two decades, the number of women with higher education and more demanding job positions has increased; as a result, marriage is being postponed [14, 16]. Higher education and higher job positions also appear to be related to economic and social independence. Given this background, marriage as a source of economic and social security has lost its importance.

Despite the novelty of the results, the following limitation cautions against any overgeneralization of the results. Lack of cooperation of some women in the interview was a problem in this study, though the researcher tried to encourage them to participate by establishing appropriate communication and explaining the confidentiality of information.

It is suggested that more studies, including quantitative work, be done to identify the basic needs of never-married women. In this situation, identifying the essential needs of these women and communicating them to the policymakers and planners in the country holds out the prospect of more facilities being established to improve the lives of never-married women. In Iran, there is no basic infrastructure to support single people in old age. As the number of these women is increasing steeply, this issue needs to be brought to the attention of policymakers.

## Conclusion

The study revealed that Iranian never-married women over 35 used various coping mechanisms to meet their needs in terms of sexual fulfillment (having sex, masturbation, and abstinence), emotional comfort (getting used to being alone, residing with family or friends), and changing lifestyle (deep believe to God’s destiny, try a lot to look beautiful and healthy and put all the time in the education and job). It is still unclear if being single is the cause or result of their current condition given the cross-sectional nature of the study. The main issue is that while some women find it easy to cope to being single, others find it challenging. To assist women in coping with singleness and living their lives in a creative, proactive, and positive way, counseling centers should be considered.

## Data Availability

The dataset generated and analyzed during this study contains the interviewee's responses and these could disclose the identity of participants. Because of the confidential nature of information, and especially given that the challenges examined in this study are critical in many countries including Iran, the dataset is not publicly available. But upon reasonable request, we can transfer your interest to the corresponding author.
